# The Long-Term Efficacy of Computed Tomography-Navigated Total Hip Arthroplasty: An 18-Year Follow-Up Study

**DOI:** 10.3390/jcm13051374

**Published:** 2024-02-28

**Authors:** Norio Imai, Dai Miyasaka, Shinya Ibuchi, Keishi Kimura, Yuki Hirano, Yoji Horigome, Hiroyuki Kawashima

**Affiliations:** 1 Division of Comprehensive Musculoskeletal Medicine, Niigata University Graduate School of Medical and Dental Sciences, Niigata 951-8510, Japan; 2 Department of Orthopedic Surgery, Niigata Bandai Hospital, Niigata 950-0909, Japan; 3 Department of Orthopedic Surgery, Uonuma Kikan Hospital, Minami Uonuma 949-7302, Japan; 4 Division of Orthopedic Surgery, Department of Regenerative and Transplant Medicine, Niigata University Graduate School of Medical and Dental Sciences, Niigata 951-8510, Japan

**Keywords:** long-term survival, total hip arthroplasty, computed tomography-based navigation system, dislocation, loosening

## Abstract

Backgroumd: There have been few reports on the long-term survival of computed tomography (CT)-navigated total hip arthroplasty (THA), which should lead to a lower incidence of dislocation and loosening. In this study, we examined survivorship, dislocation, and loosening incidence using plain radiographs over a minimum 15-year follow-up after CT-navigated THA.Methods: We retrospectively reviewed 145 consecutive CT-navigated THAs for >15 years. We surveyed the angles placed in both the acetabular and femoral components, survivorship, the occurrence of dislocation, the revision rate, and the fixation grade of the acetabular component. Results: The mean follow-up duration was 18.4 years. Overall, 73.8% of THAs were within the safe zone of Lewinnek. There were four dislocations (2.8%), with three occurring within 1 month after surgery and the other within 7 years after surgery. Revision THA was performed in one case (0.69%); consequently, the survival rate was 99.3%. The fixation grade was evaluated in 144 hips, and those were evaluated as having “no loosening”. Conclusions: CT-navigated THA was speculated to contribute to long-term survivorship, with a low rate of loosening, even after 18 years of follow-up. It was speculated that the acetabular component was placed at an acceptable insertion angle and a suitable position for stable initial fixation.

## 1. Introduction

Accurate implantation in total hip arthroplasty (THA) is important for long-term survivorship following THA [1]. Impingement between the femoral neck and edge of the acetabular component after THA leads to dislocation and/or cup loosening in the early phase [2,3]. A highly inclined and/or anteverted cup increases the edge loading, leading to a risk of wearing, even in highly crosslinked polyethylene [4]. Thus, physicians have used computed tomography (CT)-based navigation to achieve more precise implantation in THA [3,5,6]. It has been reported that a more precise placement of the acetabular component should decrease the incidence of dislocation and lessen the loosening, consequently leading to longer survivorship [1]. Sugano et al. [1] reported that survivorship following THA with a CT-based navigation system was similar to that without a CT-based navigation system, although the dislocation rate decreased by an average of 13 years of follow-up. However, the long-term survival of CT-navigated THA for >15 years has not been investigated previously. Therefore, in this study, we surveyed survivorship, the incidence of dislocation, and loosening using plain radiographs at a minimum 15-year follow-up after THA. We hypothesized that the survival rate would be good even for >15 years after THA because of the precise insertion of the acetabular component using a CT-based navigation system.

## 2. Materials and Methods

### 2.1. Ethics Statements

The study design was approved by the Institutional Review Board of our university (details blinded for peer review). The requirement for informed consent was waived because this was a cross-sectional, retrospective, non-interventional study. This study was performed in accordance with the relevant guidelines and regulations.

### 2.2. Study Design and Population

We retrospectively reviewed consecutive patients who underwent THA at our institution between 1 October 2002 and 30 September 2007. Patients with missing follow-up data, such as those who transferred to a different hospital, those who dropped out due to poor physical condition or at their discretion, and those whose data were collected for <15 years were excluded.

**Figure 1:**
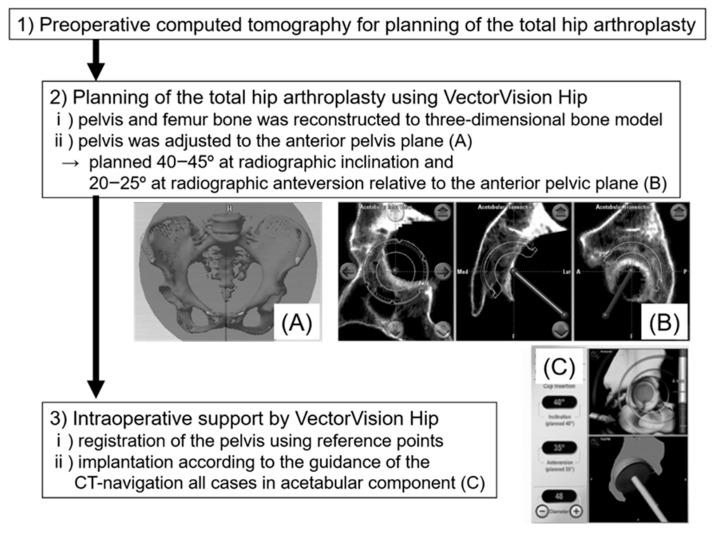
Navigation process from planning to intraoperative support. (**A**) Adjustment of anterior pelvic palne. (**B**) Planning of the acetabular component. (**C**) Intraoperative support of CT-based navigation system.

We planned an implantation angle of 40–45° of radiographic inclination and 20–30° of operative anteversion relative to the anterior pelvic plane (APP) (Figure 1) [7]. The anteversion of the femoral component was aimed at 10–30° relative to the retrocondylar plane (RCP), which consisted of the most posterior point of both posterior condyles and that of the greater trochanter [8], to avoid implant impingement as a cause of dislocation following THA. A 10° lipped polyethylene liner was used if dislocation or impingement occurred easily during trial reduction. All surgeries were performed by four experienced surgeons using direct lateral, anterolateral supine, or Orthopädische Chirurgie of München approach with patients under general anesthesia. All acetabular components were inserted under the navigation system to guide the cup insertion angle and position, such as height and depth, according to CT-based three-dimensional (3D) preoperative planning (Figure 1). Concerning the insertion of the femoral component, the navigation system could support the femoral neck osteotomy level and stem anteversion; however, it was not used in all cases during this study period. Osteophytes around the acetabulum were resected after cup placement when bony impingement occurred between the femurs.

The uncemented acetabular component Trilogy^®^ (Zimmer Biomet, Warsaw, IN, USA) was inserted via CT-based navigation system guidance (VectorVision Hip^®^, Brainlab AG, Munich, Germany) and highly crosslinked polyethylene (Longevity^®^, Zimmer Biomet, Warsaw, IN, USA). The femoral component was chosen from three kinds of implants, namely the VerSys Midcoat collarless stem^®^, Fiber metal taper^®^, and Cemented^®^ (Zimmer Biomet, Warsaw, IN, USA), adjusted to the configuration of each femur. The diameters of the femoral heads, made of zirconium, were 22, 26, or 28 mm.

### 2.3. Data Collection

We used ZedView^®^ software (Version 15.0; Lexi, Tokyo, Japan) to measure the implantation angle, such as the inclination and anteversion of the acetabular component and the anteversion of the femoral component, after creating a 3D bone model reconstructed from CT images examined about one week after their surgery [9,10]. We examined each limb using a multi-slice CT scanner (Aquilion 64TM, Toshiba Medical Systems, Otawara, Tochigi, Japan) with about 600 slices of 1.25 mm thickness in each limb. To measure the inclination and anteversion of the acetabular component, we adjusted the pelvis model to the APP [7]. We also adjusted the 3D femur model to the RCP [8]. We also evaluated the percentage within the safe zone described by Lewinnek et al. [7], both within 30–50° of radiographic inclination and 5–25° of radiographic anteversion.

### 2.4. Follow-Up

We followed up with the patients at 1, 3, 6, and 12 months after surgery and annually thereafter. We examined the modified Harris Hip Score (mHHS) [11], and an anteroposterior plain radiograph of both hips was obtained at each follow-up. We evaluated the mHHS before surgery and at the time of the last visit to our institution. We also evaluated the fixation grade of the acetabular component on plain radiographs using the DeLee and Charnley classification [12].

We surveyed the occurrence and duration of dislocation after surgery, revision rate, cause, and the duration of revision after surgery. We considered revision as the presence of displacement of the components by 10° or >3 mm on plain radiographs or loosening with symptoms such as pain, discomfort, or recurrent dislocation with interference in daily living.

### 2.5. Data Analyses

The difference in mHHS before and after THA at the final visit was compared using the paired *t*-test. Moreover, intraclass correlation coefficients (ICCs) were applied to evaluate the intra-rater and inter-rater reliabilities. Intra-rater reliability was evaluated by one observer (S.I.) and measured twice at intervals of more than one week. Inter-rater reliability was evaluated by another observer (N.I.) and compared with the measurement between the two observers. SPSS (version 28; IBM Corp., Armonk, NY, USA) was used for statistical analyses.

## 3. Results

**Figure 2:**
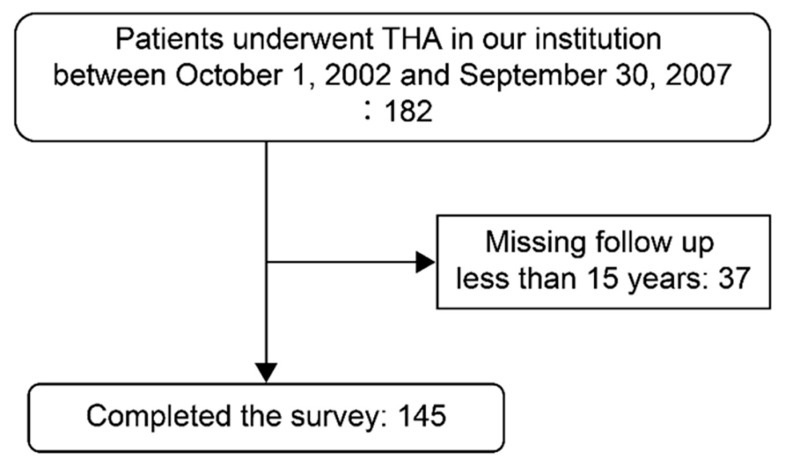
Flow diagram of the participant selection process.

**Figure 3:**
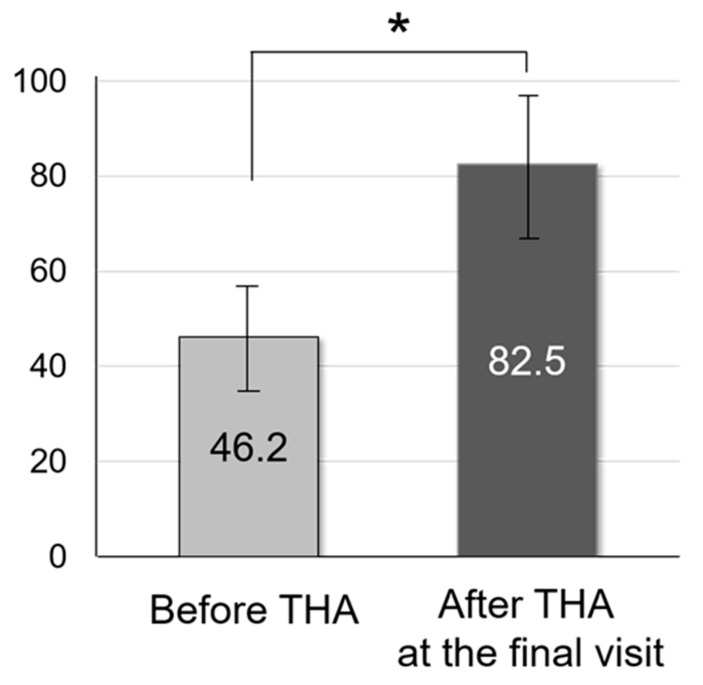
Comparison of modified Harris Hip Score before and after total hip arthroplasty. * Modified Harris Hip Score was significantly improve after THA.

Of 182 THAs, 37 hips were excluded owing to missing follow-up data for <15 years; consequently, we included 145 hips: 131 in 108 women and 14 hips in 12 men (Figure 2). The mean age at operation was 57.3 years (Table 1). Details of the study participants are presented in Table 1. The mean follow-up duration was 18.4 years. mHHS was significantly improved after THA (Figure 3).

**Figure 4:**
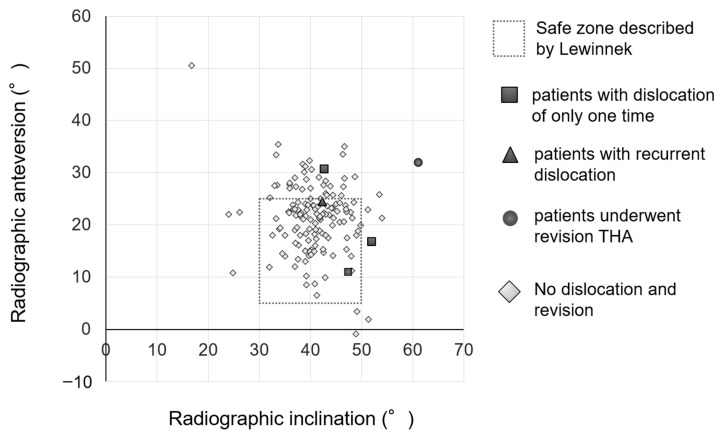
Scatter plot of radiographic inclination and radiographic anteversion of the acetabular component. Overall, 74% of total hip arthroplasties were within the safe zone of Lewinnek.

The numbers and types of implants used in our survey period are described in Table 1. Radiographic inclination and anteversion measured using ZedView (Version 15.0; Lexi, Tokyo, Japan) were 41.0 ± 5.8° and 21.3 ± 6.9° relative to the APP, respectively (Figure 4). Overall, 107 of the 145 cases (73.8%) were within the safe zone of Lewinnek (Figure 4). Stem anteversion was 19.4 ± 13.9° relative to the RCP. The mHHS significantly improved from 46.2° before THA to 82.4° at the final visit (*p* < 0.001).

There were four dislocations (2.8%) during the survey period (Figure 4). Three dislocations occurred within 1 month after surgery, whereas the other dislocation occurred 7 years after surgery. All four cases with dislocation were manually reduced without any surgery. These four cases were performed using a direct lateral approach.

Revision of THA was needed in one case at 4 months after THA (0.69%); consequently, the survival rate was 99.3%. In this case, the acetabular component began to tilt at 1 month postoperatively, and the inclination on plain radiography increased by >10° at 4 months after THA. We considered this to be due to the safety of the initial fixation. There was one case of recurrent dislocation, in which the initial dislocation occurred 7 years after THA, as aforementioned. In this case, the age of the patient who underwent primary surgery was 76 years, and dislocation occurred approximately every 2–3 years; therefore, the patient did not interfere with daily life and did not wish to undergo reoperation.

**Figure 5:**
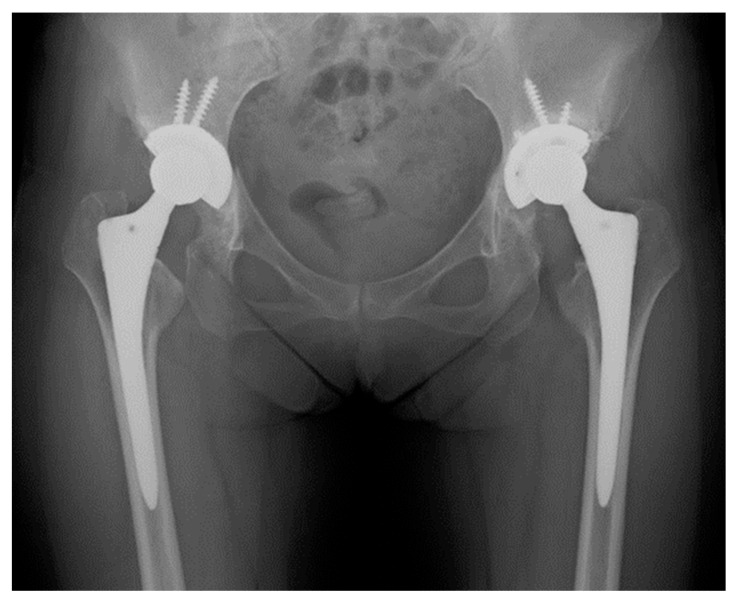
Plain X-ray of a 76-year-old woman, 15 years after THA; we evaluated this case as grade IA (no loosening) on the right side and as grade IB (radiolucent line was observed only in the outer one-third area) using the DeLee and Charnley classification.

The fixation grade of the acetabular component was evaluated using the DeLee and Charnley classification [12] for 144 hips. One was excluded because it loosened 4 months after surgery. One hundred forty components (97.2%) were evaluated as grade IA (no loosening) and three as grade IB (2.1%) (the radiolucent line was observed only in the outer one-third area) (Figure 5). There were no components evaluated as grade IC, II, or III; therefore, no component was evaluated as “unstable fixation”. The reliability of the measurement values was high, and all intra-observer and inter-observer ICCs were >0.9 (Table 2).

## 4. Discussion

In this study, we found that the survivorship of CT-navigated THAs was >99%, with stable fixation over an 18-year follow-up, which supports our hypothesis.

CT-navigated THA can result in a more precise placement closer to preoperative planning than conventional methods [5,10,13,14]. This was speculated to decrease the incidence of dislocation and loosening, consequently leading to longer survivorship; however, these effects have not been reported.

We confirmed that CT-based navigation contributed to placing the acetabular component with high precision (Figure 2). Previous reports have described the correlation between precise cup implantation and clinical benefits; however, many of the cases were followed up for a short period [15,16]. In our current study, the survival rate was 99.3% when the endpoint was revision surgery over a >18-year follow-up. Long-term survivorship was previously reported as 100% over a 13-year follow-up in Sugano et al.’s study [1]. Our finding was similar to their result, although our survey had an approximately 5-year longer follow-up period. As far as we are aware, there are few reports of long-term results following THA without CT-based navigation performed during the same period. In previous studies, the revision rate was 4.4% on ceramic-on-ceramic implants in a 13-year follow-up [1] and 3.5% on the same implant in a 9-year follow-up [17]. Therefore, THA with CT-based navigation may improve long-term survivorship. Moreover, the loosening of the acetabular component was observed in only 2.1% of cases. It was speculated that the acetabular component was placed not only with an acceptable insertion angle, such as inclination and anteversion, but also in a suitable position, such as at the center of the anteroposterior walls and/or with enough coverage of the acetabular wall for stable initial fixation owing to the support of the CT-based navigation system.

During our survey period, there were four dislocations. The dislocation rate following THA seemed to be low during the study period (4–8%) [1,18,19,20], although it was slightly higher than that reported in recent years (approximately 1%) [10,21]. The safe zone of Lewinnek [7] was widely used at that time; however, more detailed safe zones, such as the concepts of combined anteversion theory [22,23] and functional pelvic plane [24] for the functional orientation of the acetabular component, were not yet prevalent; therefore, whether our planning was correct remains controversial.

Various factors contribute to dislocation. Moreover, dislocation occurs not only due to the implantation angle of the acetabular component but also due to the anteversion of the femoral component, head size, neck shaft angle of the femoral component, the tension of the soft tissue, the preservation of the attachment of the muscle and/or joint capsule, and surgical approach [10,25,26]. In our study period, femoral head size was 22, 26, or 28 mm, which is smaller than those in recent years and may have affected the dislocation [16,27]. Moreover, the postoperative patient guidance, including the prohibited hip joint position, may not have been consistent [28].

This study had several limitations. We did not compare navigation and non-navigation groups. Thus, we could not demonstrate the superiority of CT-navigated THA compared to a non-navigated THA in this study. We can merely state that CT-navigated THAs seemed to perform as well as non-navigated THAs. To actually illustrate a difference, if one did exist, a randomized, prospective, multi-center study would need to be performed over a number of years with several thousand patients in each group to ensure that it is sufficiently well powered to reach a statistically significant conclusion. For femoral component placement, we did not use support from a CT-based navigation system for all THAs. However, variability in femoral neck anteversion can affect the range of motion and hip stability when combined with anteversion [23]. In this study, dislocation was 2.8%; especially, dislocation in the early phase after THAs might have been due to the malpositioning of acetabular and/or femoral components in three cases (2.1%). Therefore, we believe that our placement of the femoral component was acceptable even though there were several variations in this placement. We planned and placed the femoral components according to the safe zone described by Lewinnek et al. [7] relative to the APP at that time. Recently, pelvic tilt has also been considered for the insertion angle [24]; thus, this safety zone is not always considered to be correct. Finally, THAs in this study period were performed by four surgeons, and they were all well experienced. Therefore, the outcomes may differ if these factors are considered. Further examination is required; for instance, a comparison can be performed between a group with THA supported by CT-based navigation and a group without navigation with a unified implantation concept such as that established by Widmer et al. [22] and a longer survey duration.

## 5. Conclusions

CT-navigation system was speculated to contribute to long-term survivorship after THA, with a low rate of loosening even after 18 years of follow-up. It was speculated that the acetabular component was placed not only at an acceptable insertion angle but also at a suitable position for stable initial fixation.

## Figures and Tables

**Table 1:** Participants’ characteristics.

Age (Years)	57.3 ± 9.6
Sex (men/women)	14/131
Right/left hip	69/76
Follow-up period (years)	18.4 ± 1.7
Body mass index (kg/m^2^)	23.4 ± 3.1
Surgical approach	Direct lateral, 109 Anterolateral supine, 11 Orthopädische Chirurgie of München approach, 25
Cause	Hip osteoarthritis, 117 Idiopathic osteonecrosis of the femoral head, 21 Rheumatoid arthritis, 6 Pigmented villonodular synovitis,
Acetabular component	Trilogy^®^, 145
Femoral component	VerSys Midcoat collarless stem^®^, 89 Fiber metal taper^®^, 48 Cemented^®^, 8
Head size (mm)	22, 7 26, 103 28, 35

**Table 2:** Intra-rater and inter-observer reliabilities of the measurement values.

	Intra-Observer Reliability				Inter-Observer Reliability			
	MAD	ICC	95% CI	*p*-Value	MAD	ICC	95% CI	*p*-Value
Radiographic anteversion (°)	1.4 ± 0.8	0.971	0.962–0.978	<0.001	1.9 ± 1.4	0.932	0.912–0.945	<0.001
Radiographic inclination (°)	1.3 ± 0.9	0.974	0.963–0.980	<0.001	2.3 ± 1.8	0.941	0.922–0.956	<0.001
Stem anteversion (°)	1.6 ± 1.6	0.956	0.934–0.978	<0.001	2.2 ± 2.1	0.950	0.933–0.962	<0.001

MAD, mean absolute difference; ICC, inter-class correlation coefficient; CI, confidence interval.

## Data Availability

No new data were created or analyzed in this study. Data sharing is not applicable to this article.

## Abbreviations

THA, total hip arthroplasty; CT, computed tomography; APP, anterior pelvic plane; RCP, retrocondylar plane; 3D, three dimensional; mHHS, modified Harris Hip Score; ICCs, intra-class correlation coefficients.
